# Supplementary material for: The low and high doses administration of lutein improves memory and synaptic plasticity impairment through different mechanisms in a rat model of vascular dementia
Source: PLoS One. 2024 May 15;19(5):e0302850. doi: 10.1371/journal.pone.0302850 (PMC11095768; doi:10.1371/journal.pone.0302850)
Supplement: Attachment — Submitted filename: Responses to Reviewers.docx [file pone.0302850.s001.docx]

**Dear Editor and Reviewers,**

We highly appreciate the detailed valuable comments of the referees on our manuscript of ‘ONE-D-24-00251’. The suggestions are quite helpful for us and we incorporate them in line with your suggestions and those of the reviewers. The following responses have been prepared to address all comments in a **point*–*by–point** fashion and we hope the Reviewers and the Editors will be satisfied with our responses to the comments and the revisions for the original manuscript. Any change to the manuscript has been highlighted by using *track* ***changes*** colored text*.*

Journal requirements:

When submitting your revision, we need you to address these additional requirements.

1. Please ensure that your manuscript meets PLOS ONE's style requirements, including those for file naming. The PLOS ONE style templates

We have revised the manuscript to comply with PLOS ONE's style requirements.

2. We noticed you have some minor occurrence of overlapping text with the following previous publication(s), which needs to be addressed:

https://pubmed.ncbi.nlm.nih.gov/32916222/

https://onlinelibrary.wiley.com/doi/10.1002/brb3.3351

https://pubmed.ncbi.nlm.nih.gov/34116172/

In your revision ensure you cite all your sources (including your own works), and quote or rephrase any duplicated text outside the methods section. Further consideration is dependent on these concerns being addressed.

We have corrected the minor overlapping, we also would like to clarify that the overlap is confined to the methods section, and we have appropriately cited all relevant sources, the three studies are related to our team's previous research. We appreciate your attention to this matter.

3. In your Methods section, please report the source of Lutein used for your study.

Thank you, we have added the source of Lutein in method section

4. To comply with PLOS ONE submissions requirements, in your Methods section, please provide additional information regarding the experiments involving animals and ensure you have included details on (1) methods of sacrifice, (2) methods of anesthesia and/or analgesia, and (3) efforts to alleviate suffering.

We have incorporated the following details into the Methods section: To alleviate animal suffering, all animals euthanized at end of the experiment, following induction of deep anesthesia with urethane, rats were sacrificed using a guillotine. The personnel responsible for sacrifice were trained in the humane and ethical handling of animals, and the procedures were conducted in compliance with the Institutional Ethics Committee of Shiraz University of Medical Sciences.

5. We note that the grant information you provided in the ‘Funding Information’ and ‘Financial Disclosure’ sections do not match.

Thank you, The Research Council of Shiraz University of Medical Sciences, Shiraz, Iran, financially supported this study (Grant Number: 26316), as a thesis of Mrs. Asadi nejad for acquiring M.Sc. degree in physiology.

When you resubmit, please ensure that you provide the correct grant numbers for the awards you received for your study in the ‘Funding Information’ section**.**

Thank you; we have also provided an explanation of the grant information in the cover letter.

6. Thank you for stating the following in the Acknowledgments Section of your manuscript:

“The authors acknowledge the Research Council of Shiraz University of Medical Sciences, Shiraz, Iran for the financial support of this study (Grant Number: 26316), as a thesis of Mrs. Asadi nejad for acquiring M.Sc. degree in physiology.”

We note that you have provided funding information that is not currently declared in your Funding Statement. However, funding information should not appear in the Acknowledgments section or other areas of your manuscript. We will only publish funding information present in the Funding Statement section of the online submission form.

Please remove any funding-related text from the manuscript and let us know how you would like to update your Funding Statement. Currently, your Funding Statement reads as follows:

“The author(s) received no specific funding for this work.”

Please include your amended statements within your cover letter; we will change the online submission form on your behalf.

Thank you once again. We have omitted the funding information from the Acknowledgments section and have also provided a detailed explanation in the cover letter.

7. In this instance it seems there may be acceptable restrictions in place that prevent the public sharing of your minimal data. However, in line with our goal of ensuring long-term data availability to all interested researchers, PLOS’ Data Policy states that authors cannot be the sole named individuals responsible for ensuring data access (http://journals.plos.org/plosone/s/data-availability#loc-acceptable-data-sharing-methods).

Data requests to a non-author institutional point of contact, such as a data access or ethics committee, helps guarantee long term stability and availability of data. Providing interested researchers with a durable point of contact ensures data will be accessible even if an author changes email addresses, institutions, or becomes unavailable to answer requests.

Before we proceed with your manuscript, please also provide non-author contact information (phone/email/hyperlink) for a data access committee, ethics committee, or other institutional body to which data requests may be sent. If no institutional body is available to respond to requests for your minimal data, please consider if there any institutional representatives who did not collaborate in the study, and are not listed as authors on the manuscript, who would be able to hold the data and respond to external requests for data access? If so, please provide their contact information (i.e., email address). Please also provide details on how you will ensure persistent or long-term data storage and availability.

We understand the importance of data availability and appreciate the guidance provided by PLOS' Data Policy. To ensure long-term stability and availability of the data, we established a data access point within our institution. We provided contact details, including phone/email, for our data access committee with following addresses: Mrs Masoumi, phone number +987132302026 E-mail: medphyzio1@sums.ac.ir

We recognize the necessity for a durable point of contact, and we assure you that the provided information will guarantee accessibility to the data even in the case of changes in authors' contact details or availability.

Thank you for your understanding, and we provided the required non-author contact information.

8. Please review your reference list to ensure that it is complete and correct. If you have cited papers that have been retracted, please include the rationale for doing so in the manuscript text, or remove these references and replace them with relevant current references. Any changes to the reference list should be mentioned in the rebuttal letter that accompanies your revised manuscript. If you need to cite a retracted article, indicate the article’s retracted status in the References list and also include a citation and full reference for the retraction notice.

Thank you for the feedback. I have thoroughly reviewed the reference list, ensuring its completeness and correctness.

Additional Editor Comments:

Reviewer #1: The study is novel. The study is accordance with research and publication ethics. They have clearly mentioned about their methodology, ethical clearance and statistical data of evaluation and conclusion is in accordance with the aim of the study.

Thank you for sharing the positive feedback about the study. It's great to hear that the study is considered novel and aligns well with research and publication ethics. The clarity in methodology, ethical clearance, and the appropriate presentation of statistical data is crucial for ensuring transparency and reliability in research. If you have any specific questions or if there are additional aspects, you'd like us to address, please feel free to let us know. We appreciate your positive assessment.

Reviewer #2: I have had the opportunity to review your manuscript, I must commend you on your interesting investigation into the effects of lutein administration in a rat model of bilateral-carotid vessel occlusion, particularly focusing on spatial and fear memory, synaptic plasticity, and hippocampal cells. Your study addresses an important area of research, and your findings have the potential to contribute significantly to our understanding of the therapeutic potential of lutein in vascular dementia. However, I have some queries and suggestions that I believe need to be addressed before the manuscript can be considered for publication.

Thank you for your positive feedback on the study's significance and potential contribution to understanding the therapeutic effects of lutein in vascular dementia. I appreciate your diligence in reviewing the manuscript.

1. Were the doses of lutein chosen for this study based on previous research? Can you provide more details about the rationale behind choosing the doses of 0.5mg/kg and 5mg/kg for lutein administration?

The selection of lutein doses in this study was based on previous research findings and considerations related to the potential therapeutic effects of lutein. The rationale for choosing the specific doses of 0.5mg/kg and 5mg/kg is rooted in existing literature that suggests a range of effective concentrations for lutein interventions in various experimental settings. These doses were selected to encompass a lower and higher range within this effective concentration spectrum, allowing the researchers to observe potential dose-dependent effects and evaluate the optimal dosage for the intended outcomes. The decision was also influenced by safety considerations and previous studies that demonstrated beneficial effects within similar dose ranges.

2. Could you please clarify whether your study adhered to the ARRIVE guidelines for reporting animal research? Ensuring compliance with these guidelines is essential for transparency and reproducibility in animal studies.

Thank you for your comment. We have thoroughly reviewed our study and can confirm that it adheres to the ARRIVE guidelines for reporting animal research. Additionally, we would like to inform you that ethical approval was obtained, and the study was conducted in accordance with the guidelines provided by the Institutional Animal Ethics Committee under the ethical code IR.SUMS.AEC.1401.077. The revised manuscript now explicitly mentions this ethical approval to enhance transparency and compliance with ethical standards.

3. Could the authors please provide information on the age of the rats selected for this study? Clarifying the age range of the experimental animals is important for understanding the developmental stage at which the interventions were applied and interpreting the study findings accurately.

Thank you for this comment we added detail of age as follow: Adult male Sprague Dawley rats (250-300 g, aged 7-10 weeks) were housed under …

4. Were there any limitations or challenges encountered during the experimental procedures or data analysis that could have influenced the study outcomes?

A-Dosage Dependency: The study revealed that the high dose of lutein (5mg/kg) had more significant effects on passive avoidance memory, spatial learning and memory, electrophysiological measures, hippocampal cell loss, and oxidative stress compared to the low dose (0.5mg/kg). However, the optimal dosage range and potential side effects of lutein were not thoroughly explored. Future studies should investigate a wider range of doses to determine the dose-response relationship and potential dose-dependent effects.

B-Generalization to Human Population: The experiment was conducted on 2-vessel occlusion (2VO) rats, and the findings may not directly translate to humans. Animal models have inherent differences from human physiology, and caution should be exercised when extrapolating these results to clinical applications. Further research, including human clinical trials, is needed to validate the relevance of these findings in humans.

C-Short-term Follow-up: The experiment assessed the effects of lutein treatment over a relatively short-term period. Long-term effects and potential sustained benefits or side effects of lutein administration were not investigated. Future studies should consider longer follow-up durations to better understand the persistence and durability of the observed effects

5. Based on the findings of this study, what are the potential implications for future research or clinical applications related to lutein administration in vascular dementia (VD) patients?

Thank you for this good comment, we are in the process of designing and planning a clinical trial to investigate the effect of lutein on individuals suffering from vascular dementia: Given that the study was conducted on 2-vessel occlusion (2VO) rats, the translation of these findings to human patients with vascular dementia warrants further investigation. Conducting well-designed clinical trials involving individuals with vascular dementia will be essential to assess the efficacy and safety of lutein in a clinical setting.

Long-term Effects and Safety Profile: The study primarily focused on short-term effects, and the long-term effects of lutein administration were not thoroughly explored. Future research should investigate the sustained benefits, potential side effects, and safety profile of lutein over extended treatment periods.

Comparison with Existing Treatments: Comparative studies assessing the efficacy of lutein in comparison to existing treatments for vascular dementia could provide valuable insights. Understanding how lutein compares to standard treatments or other potential interventions will contribute to its potential integration into clinical practice.

6. PLOS authors have the option to publish the peer review history of their article (what does this mean?). If published, this will include your full peer review and any attached files.

Thank you, yes!
